# Editorial: Inherited Protein Glycosylation Defects in Humans

**DOI:** 10.3389/fgene.2022.851438

**Published:** 2022-03-14

**Authors:** Aleksandra Jezela-Stanek, Karolina M. Stepien, Anna Tylki-Szymanska

**Affiliations:** ^1^ Department of Genetics and Clinical Immunology, National Institute of Tuberculosis and Lung Diseases, Warsaw, Poland; ^2^ Adult Inherited Metabolic Disorders, Salford Royal NHS Foundation Trust, Salford, United Kingdom; ^3^ Department of Pediatric Nutrition and Metabolic Diseases, Children’s Memorial Health Institute, Warsaw, Poland

**Keywords:** congenital disorder of glycosylation, CDG, glycosylation, deglycosylation, GPIBD

Facilitated by the wide availability of next-generation sequencing-based genetic testing such as whole exome sequencing (WES) or whole genome sequencing (WGS), the number of new diagnoses within rare diseases is constantly growing. Specifically, this editorial refers to inherited metabolic diseases resulting from defects in protein glycosylation, including congenital disorders of glycosylation (encompassing N-glycosylation, O-glycosylation, multiple glycosylation), disorders of glycosphingolipids, glycosylphosphatidylinositol (GPI)-anchored protein defects (GPIBDs), and deglycosylation disorders (CDDGs).

Congenital disorders of glycosylation (CDG), first reported in the medical literature in 1980 by Prof. Jaak Jaeken and colleagues (Jaeken, 2010), is a group of ∼140 rare genetic, metabolic disorders resulting from defects in a complex chemical process known as glycosylation. This process involves myriad different genes, encoding a variety of proteins (called glycoproteins), such as enzymes and glycolipids, which have numerous important functions in nearly all tissue types (Jaeken et al., 1980). Consequently, CDG can affect any part of the human body and a predominant neurological deterioration is a frequent manifestation of the clinical spectrum (Paprocka et al., 2021). Because CDG can be associated with a multitude of symptoms and varies widely in severity, achieving a diagnosis based on clinical criteria is challenging. Molecular genetic testing offers the greatest accuracy toward pinpointing an underlying cause.

The goal of the “*Inherited Protein Glycosylation Defects in Human Diseases*” Research Topic was to raise awareness of and improve our understanding of genetic diseases resulting from defects in proteins glycosylation. We aimed to present a comprehensive picture of these disorders, ranging from: 1) clinical manifestation (in reference to specific internal organs), 2) genetic background, 3) underlying pathomechanisms, and 4) discussion of treatment options. The final topic issue has 14 published articles.

Contrary to a classical approach, wherein diagnostic analysis proceeds from phenotype to genotype, researchers are increasingly employing reverse genetics strategies, where the analysis proceeds from genotype to phenotype. As a consequence, reaching a genetic diagnosis is only the beginning of the clinical journey and can open up a plethora of complex considerations. For CDGs, GPIBDs, and CDDGs, geneticists and metabolic physicians face perpetual challenges regarding prediction of clinical manifestations, availability of natural history and/or availability of any treatment options. Consequently, and to overcome these challenges, a new genetic diagnosis can generate new basic science and clinical research opportunities, spark international collaboration and encourage researchers to share experience within the wider medical community. Furthermore, advocacy from patient organisations offer complementary advances toward improving diagnosis, shortening the lengthy diagnostic odyssey and decreasing mortality from these rare conditions (González-Domínguez et al.).

Despite the advent of robust diagnostic technology, there are still regional limitations to WES and WGS access that are influenced by the local health economy. Given that rapid diagnosis can be hampered by poor access to specialist laboratories and tertiary metabolic centres, Lipinski and Tylki-Szymanska outlined diagnostic algorithms for a rapid and accurate diagnosis of CDGs in children based on their own clinical experience in Poland. Kosinski et al., however, outlined the difficulties of diagnosing a CDG prenatally, which highlights the need for including these disorders in the diagnostic paradigm of fetuses with certain ultrasound findings, such as diaphragmatic hernia.

Systematic employment of WES/WGS has uncovered hitherto unappreciated phenotype expansions of CDG. In one instance, liver involvement was observed in a congenital disorder of deglycosylation (Lipinski et al.), although it was not implicated previously as a core clinical feature. Clinical spectra of particular diseases were revisited; for example, Kamarus Jaman et al. emphasized ophthalmological abnormalities including early-onset retinal dystrophy and optic nerve hypoplasia as key clinical diagnostic features of SRD5A3-CDG in patients with a multisystem disorder with variable symptoms evolving over time. DOLK-CDG caused by a defect in dolichol kinase in which the congenital skin phenotype (often ichthyosis) is associated with variable extracutaneous features such as dilatated cardiomyopathy, epilepsy, microcephaly, visual impairment, and hypoglycemia are now shown to have a fatal course (Komlosi et al.). Other conditions such as PIGT and phosphomannomutase 2 deficiency (PMM2-CGD) have been better defined. The comprehensive analysis of a large cohort of both previously published and newly studied individuals with *PIGT* variants analysed by Bayat et al. broadens the phenotypic spectrum and presents evidence that p.Asn527Ser and p.Val528Met variants are associated with a mild phenotype and less severe outcomes, and *PIGT* may be considered as a new candidate gene for myoclonic, atonic epilepsy. Mercedes Serrano reported stroke-like episodes (SLE) as a manifestation of an expanded phenotype of PMM2-CDG. The characteristic but non-specific constellation of symptoms including migraine, focal neurologic deficits, hemispheric slow electroencephalogram (EEG) trace and refractory hyperpyrexia without laboratory signs of infection, are now shown to be suggestive of PMM2-CDG disorder.

Another recent report has shown expansion of the genotypic and phenotypic spectrum of another CDG. Abuduxikuer and Wang present four new cases affected with a previously unreported deleterious variant causing SLC35A2-CDG, and describe clinical complications of the condition in pregnancy (hypothyroidism and oligohydramnios). Taken together, the evolving clinical spectrum of CDGs makes it difficult to predict long-term prognosis and clinical outcomes in adulthood, therefore there is a need for long-term follow-up of these patients with multi-disciplinary specialists.

The prevalence of other CDG types remains unknown. To address this knowledge gap, Pajusalu and colleagues report the estimated prevalence of different N-linked protein glycosylation defects calculated from population allele frequencies (Pajusalu et al.). Although the CDG group involves at least 137 defects, they included only 27 autosomal recessive protein N-glycosylation affecting defects and excluded abnormalities that affect multiple glycosylation pathways. If all 27 defects are considered, the combined prevalence of CDGs in non-Finnish Europeans is 1:22,000. If FUK-CDG and MAN2B2-CDG are excluded (lack homozygous loss-of-function (LoF) variant carriers in Genome Aggregation Database (gnomAD)), the prevalence among Europeans is slightly lower and does not exceed one in 24,000.

Apart from CDGs, this Research Topic describes the prevalence and clinical manifestations of other inherited protein glycosylation defects such as POMT2-related defective O-mannosylglycosylation of α-dystroglycan (Chen et al.) and GM1-gangliosidosis caused by the reduced activity of β-Galactosidase which leads to the accumulation of both GM1 ganglioside and also its derivative, GA1, primarily in lysosomes of neuronal tissue (Nicoli et al.).

Beyond diagnostics, this Research Topic also discusses therapeutic options. Cellular models are the first step to the new therapeutic advances. In their original research, Sumya et al. described the cellular model for Conserved Oligomeric Golgi, an octameric protein complex that orchestrates intra-Golgi trafficking of glycosylation enzymes. Nicoli et al. described various experimental therapies for GM1-gangliosidosis tested on human cell lines. Understanding the cellular pathophysiology underlying this disease is essential toward developing therapeutic avenues. Mouse models have been instrumental for the pre-clinical testing of multiple therapies for GM1-gangliosidosis.

Recent therapeutic advances aimed both at the causative defect and secondary disease manifestations in CDGs were discussed by Park and Marquardt. Unfortunately, therapies are currently limited to only a few CDG subtypes and there is no strong evidence of their efficacy due to the lack of randomized controlled trials.

In conclusion, despite an improved understanding of pathophysiology of CDG, diagnosis within this disease group remains a challenge. However, an emergent availability of genome-editing techniques is opening a promising future for CDG patients who have received a molecular diagnosis. Therefore, the focus of future research should be on documenting long-term outcomes of adults affected with these conditions, and targeted therapeutic developments to attenuate symptoms.
